# Editorial Comment to Robot‐assisted radical nephrectomy and inferior vena cava tumor thrombectomy: Initial experience in Japan

**DOI:** 10.1002/iju5.12425

**Published:** 2022-02-17

**Authors:** Kiyoshi Takahara, Ryoichi Shiroki

**Affiliations:** ^1^ Department of Urology School of Medicine Fujita Health University Toyoake Aichi Japan

**Abbreviations & Acronyms**
IVC: inferior vena cava
RA‐RN: robot‐assisted radical nephrectomy
RCC: renal cell carcinoma
RN: radical nephrectomy
TT: tumor thrombectomy

Minimally invasive surgery using laparoscopic techniques in the treatment of RCC with an IVC tumor thrombus has always been challenging; therefore, open surgery remains the standard treatment. In the context of urological procedures, RN with IVC TT, especially RA‐RN and IVC TT (RA‐RN/IVCTT), is of the most complex procedures for urologists. In addition, its safety and feasibility have not yet been established owing to the lack of literature. However, in a systematic review of 14 retrospective studies, Lardas *et al*., concluded that surgical management of patients with non‐metastatic RCC with IVC thrombus is complex, but potentially curative and acceptable.^1^ Surgical procedures in RA‐RN/IVCTT vary depending on the level of thrombus; recently, Seetharam *et al*., reported that RA‐RN/IVCTT is feasible and safe for level I, II, and III thrombus in high volume centers.^2^ Due to the high levels of surgical complexity and variation, RA‐RN/IVCTT is currently performed solely by well‐experienced surgeons in limited high‐volume centers, and its safety is still unknown.

In addition, RA‐RN is yet to be approved by the health insurance system in Japan, preventing performance of RA‐RN/IVCTT. In the present article, the authors described the first experience of RA‐RN/IVCTT,^3^ which was performed on a patient with RCC and a level I IVC by an experienced surgeon. The operation was successfully completed with a purely robotic procedure; no significant complications occurred, and perioperative outcomes were satisfactory. This article described an experience of RA‐RN/IVCTT for a RCC patient with a level I IVC thrombus, aiding improvements in understanding of the procedure’s safety and feasibility. The findings have potential novelty, especially in Japan.

## Conflict of interest

The authors declare no conflict of interest.
